# Effect of progressive pedometer based walking intervention on quality of life and general well being among patients with type 2 diabetes

**DOI:** 10.1186/s40200-014-0110-5

**Published:** 2014-11-29

**Authors:** Ruchika Guglani, Shweta Shenoy, Jaspal Singh Sandhu

**Affiliations:** Faculty of Sports Medicine and Physiotherapy, Guru Nanak Dev University, 143005 Amritsar, India

**Keywords:** Type 2 diabetes, Pedometer, Quality of life, Wellbeing, Walking

## Abstract

**Background:**

To determine the effectiveness of two goal setting pedometer based walking program for people with type 2 diabetes, one employing supervised exercise group with pedometer and the other employing self reported group with pedometer.

**Methods:**

A total of 102 type 2 diabetic outpatients (28 women, 74 men) between the age of 40-70 years were recruited and randomly allocated into 3 groups: supervised exercise group with pedometer (Group A), self reported exercise group with pedometer (Group B) and a control group (Group C) for 16 weeks. Subjects were asked to respond to the Audit of Diabetes Dependent Quality of Life (ADDQoL) and well being questionnaire at two occasions i.e. 0 week and after 16 weeks of intervention. Paired t test were used within the groups to compare Mean ± SD for all the parameters at baseline and at the end of 16 weeks. Differences between the groups were compared using analysis of variance (ANOVA). Statistical difference was further analyzed by Post hoc analysis using Bonferroni method.

**Results:**

The item “Freedom to eat” had the highest negative impact among all the subgroups. Other domains that were adversely affected by diabetes are ‘leisure activity’, ’do physically’, ‘physical appearance’, ‘self confidence’, ‘future’ and ‘financial situation’. In the group A significant reduction were noted among all the items except long distance journey (p<0.05). In the group B participants experienced reduction among all the domains except long distance journey, sex life and living condition.

**Conclusion:**

Pedometer determined activity has the potential to improve the quality of life. Supervised Walking using a pedometer was found more effective in improving quality of life and general wellbeing for Asian Indians with type 2 diabetes.

**Clinical trial registry India (CTRI):**

[CTRI/2012/10/003034].

## Background

Diabetes is one of the most debilitating lifestyle disease affecting millions of people worldwide. Type 2 diabetes mellitus (T2DM) has become a major worldwide problem with an exponential rise in numbers in recent decades. Currently there are 285 million people with diabetes worldwide that are set to increase up to 438 million by 2030 i.e. a 54% increase [1]. T2DM is an epidemic affecting millions of people worldwide constituting nearly 90% of the diabetic population in any country [2]. Factors contributing to the rapid increase in the diabetes burden are population growth, aging, urbanization and increasing prevalence of obesity and physical inactivity [3].

Diabetes is a psychologically demanding chronic disease, with psychological factors pertinent to nearly every aspect of the disease and treatment [4]. Quality of life is an important outcome in its own right, as it may influence the patient’s self care activities, which may consequently impact their diabetes control and management [5]. In the medical domain it denominates aspects of health from the patient’s point of view and could better be expressed as “subjective health” or “functional status and wellbeing” [6]. Diabetes may affect variety of aspects of quality of life that includes never-ending demands of diabetes care, such as eating carefully, exercising and monitoring blood glucose [7]. In addition to diabetes related complications, episodes of hypoglycaemia, fear of hypoglycaemia, changes in the life style and fear of long term consequences may too worsen the quality of life.

Understanding dimensions of quality of life, which are associated with co morbidities of diabetes and depression, is important for day-to-day clinical management. Knowledge of these dimensions also helps to aide public health policy initiatives, aimed at improved health outcomes for T2DM populations [8]. With this global burden, it is important to manage and control diabetes to prevent development of complications [9], as quality of life is likely to be a priority over quantity of life [10]. The risk of cardiovascular disease can be reduced by an estimated 35% to 55% through adoption and maintenance of an active life style [11].

Literature has shown that physical activity and exercise can have a significant impact on both treating and preventing or delaying the onset of T2DM. Walking interventions can be effective in reducing body weight, body mass index (BMI), waist and hip circumference, body fat, blood pressure, cholesterol-HDL (High-density lipoprotein) ratio and may also be effective in improving mood and quality of life [12]. Pedometer based physical activity programs provide an objective measure of patient’s behaviour for evaluating intervention effects, they also provide ready feedback to individuals for behavioural goal setting and monitoring progress towards those goals [13]. The step counting function of pedometers can also be used to motivate individuals to increase physical activity, especially when they are encouraged to record daily step counts and set specific step count goals [14]. Taking 10,000 steps/day appears to be a reasonable target of daily activity for healthy adults and several studies have documented the health benefits of attaining these levels [15].

Physiological parameters traditionally measured in T2DM patients like glycaemia, glycated haemoglobin, blood pressure, cholesterol and weight was not enough to evaluate diabetes quality of life for which specific quality of life parameters are preferred [16]. The Audit of Diabetes-Dependent Quality of Life (ADDQoL) is a disease-specific quality of life measure, which is increasingly being used to examine the patient’s perception of the impact of diabetes on their quality of life [17]. The ADDQoL was designed to include life domains which may be impacted by diabetes [18]. ADDQoL19 is a 19 item disease specific instrument designed to measure individual perception of the impact of diabetes on quality of life and this instrument has been validated by Prof. Bradley, (Royal Holloway- University of London) for Asian Indians [5].

Against this background the aim of the present study was to determine;

1. The influence of physical activity intervention on quality of life, in individuals with type 2 diabetes.
2. Improvement in quality of life due to motivation from pedometer use.

## Material and methodology

The prospective randomized controlled trial study was conducted in the department of sports medicine and physiotherapy, Guru Nanak Dev University, Amritsar.

### Study participants

A total of 102 outpatients with T2DM (28 women and 74 men) were recruited from Amritsar. Informed consent and baseline measurements were completed before randomization. The study was given approval by Institutional ethical committee of Guru Nanak Dev University, Amritsar (Ref. No.- 321/SMP). Subjects were recruited after the explanation of protocol and clearance from physician. Demographic information and health history were obtained from all the participants. Men and women between age of 40 to 70 years were included in the study. The inclusion criteria were as follows (i) ≥1 year diagnosis of type 2 diabetes (ii) not taking insulin (iii) no physical activity limitation (iv) were not enrolled in any other physical activity program previously or simultaneously. Exclusion criteria were disease or condition e.g. any evidence of coronary artery disease, nephropathy, uncontrolled hypertension, diabetic complications and moderate severe orthopaedic/cardiovascular/respiratory/nephropathy condition that would interfere with physical activity.

### Procedure

Participants were randomly allocated into one of the three groups by the random lottery approach: supervised exercise group with pedometer (group A), self reported exercise group with pedometer (group B) and a control group (group C). A few initial sessions of familiarization with pedometer [19] and the understanding of Borg scale [20] were given to all the participants in group A and group B. Similar instructions described by Tudor Locke et al. [21] were given to all the subjects about handling and placement of the pedometer. Use of Borg scale (Rate of perceived exertion) with target perceived intensity of moderate, somewhat hard or hard is sometimes recommended as a possible alternative to heart rate based on maximal exercise testing. RPE scales are reported as valid and reliable for assessing the level of exertion during aerobic exercise [22]. Although, RPE is a subjective measure, a person’s exertion rating may provide a fairly good estimate of the actual heart rate during physical activity [20]. Two initial sessions of physical activity for familiarization with RPE scales were given to the subjects in group A to let them understand the relation between capability of doing physical activity and required effort intensity. Subjects in group A were taught to adjust the intensity of the activity by speeding up and slowing down the speed of walking through RPE scale. No dietary modifications were advised while this intervention. Patients were advised to eat 1–2 hrs before exercise to avoid hypoglycaemia and maintain hydration levels.

Baseline readings of all the parameters for all subjects were collected before randomization. Baseline data collection was conducted for 7 days to get an estimated number of steps for group A and group B. In group A, we found approximately 3000 steps for most of the subjects in 30 minutes/session while in group B there were approximately 5000 – 6000 steps/day.

### Supervised exercise with pedometer (Group A)

In this group we aimed at achieving around 4000 steps/30-40 minutes/session under the supervision of physiotherapist. Participants walked using pedometer to achieve a target of 150 min/week moderate intensity of physical activity. Intensity of exercise was increased gradually. Subjects in group A did warm up for at least 5 minutes, keeping their target RPE in the ‘light’ range on the borg scale, then they were instructed to increase intensity up-to their target heart rate range ‘somewhat hard’ (12–14) on RPE. Subjects in group A were encouraged to increase their step counts up-to 4000/ 30–40 minutes/session and maintain it till the end of 16 weeks. All the participants were asked to report for physical activity sessions as per their schedule and were instructed to walk with pedometer under supervision for 5 days a week. A log book was maintained for all the participants. Each session took 45 – 50 minutes which included warming up and cooling down.

### Self reported exercise group with pedometer (Group B)

In this group participants were instructed to wear pedometer for 5 days in a week from “morning to night till sleeping” for 16 weeks. Tudor-Locke et al. [19], 2002 have proposed that daily steps in excess of 8000 may be roughly equivalent to the accumulation of 30 min of moderate-intensity activity on a single day. Bennett et al. [23], 2006 suggested that any 3 days (weekday or weekend) are sufficient for the reliable estimation of physical activity performed in a free-living week but in our current study we have taken pedometer based intervention and monitoring for 5 days a week. A diary was provided to all the participants so that they can record their number of steps/day. Participants were taught about the handling/working of the pedometer and were instructed to report after 16 weeks for sharing experience on change in quality of life and well being. The investigator contacted the participants on phone for their step counts. They were told to achieve target of 10,000 steps/day during intervention period without any consideration to intensity and duration. Participants could contact the researcher at any point of time for any difficulty related either to exercise protocol or the handling of pedometer. Subjects were instructed to set the pedometer to zero early morning and record the steps before going to bed.

### Control group (Group C)

Participants were asked to maintain their lifestyle and were encouraged to walk. They were not enrolled in any other intervention throughout 16 weeks. Neither pedometers nor step count data were collected.

Trial was approved by Clinical trial registry India (CTRI) [CTRI/2012/10/003034).

#### Measurements

The Audit of Diabetes Dependent Quality of Life (ADDQoL19) and Well-being questionnaire (WBQ12) was used to assess the quality of life and wellbeing in all study participants pre and post intervention. Special permission was sought from the author of the questionnaire to use the ADDQoL19 English for S. Asian (Indian) and well-being questionnaire (W-BQ12) English for India. ADDQoL 19 questionnaire includes 19 life domain specific items to be scored between (−9 to +3) depending on impact of diabetes on the quality of life. The quality of life questionnaire included a number of different life domains that may be variously impacted by diabetes and were of varying importance. The product of impact and importance of life domains is the total quality of life score of that domain. Two overview items were used, overview item 1 (OV 1) to determine generic ‘present QoL’ and overview item 2 (OV2) to determine ‘impact of diabetes on quality of life’.

#### W-BQ12

The W-BQ12 was used as an assessment tool to determine an individual’s psychological wellbeing over the past few weeks. It includes 12 items to determine general wellbeing (GWB) with subscales to measure positive wellbeing (PWB), negative wellbeing (NWB) and energy on a scale from 0 (not at all) to 3 (all the time).

#### Statistical analysis

Paired t test were used within the groups to compare Mean ± SD for all the parameters at baseline and at the end of 16 weeks. Differences between the groups were compared using analysis of variance (ANOVA). Statistical difference was further analyzed by Post hoc analysis using Bonferroni method. STATA 11.0 statistical software was used for data analysis. In this study p-value less than 0.05 has been considered as statistically significant.

## Results

The demographic characteristics of the patients with type 2 diabetes are present in Table 1. Total 102 patients were recruited and overall 88% of participants (90 of 102) completed the study. Results of the analysis are presented in Table 2.

**Table 1:** **Demographic data of study group**

	**GROUP-A (n = 35)**	**Group-B (n = 35)**	**Group-C (n = 32)**
**Age (years)**	54.4 ± 7.7	55.7 ± 8.7	50.9 ± 5.5
**Sex**	7 F, 28 M	9 F, 26 M	12 F, 20 M
**Duration of diabetes (in yrs)**	6.2 ± 2.4	5.8 ± 2.2	5.5 ± 2.1
**Height (centimetres)**	171.8 ± 7.0	170.8 ± 6.9	171.1 ± 7.2

**Table 2:** **Pre intervention and post intervention changes in quality of life domains**

**Items**	**Groups**			**ANOVA**		**Post hoc (Bonferroni)**		
	**GROUP A**	**GROUP B**	**GROUP C**	**F**	**P**	**A & B**	**A&C**	**B&C**
**1. LEISURE ACTIVITY**								
**PRE**	−3.2 ± 1.9	−3.5 ± 1.5	−3 ± 1.4	0.72	0.48	1.0	1.0	0.72
**POST**	−2.3 ± 1.19	−3 ± 1.3	−3.1 ± 1.3	5.81	0.004	0.03	0.00	1.0
**P VALUE**	0.0001	0.026	0.42					
**2. WORKING LIFE**								
**PRE**	−2.9 ± 1.3	−3 ± 1.4	−2.7 ± 1.3	0.30	0.73	1.0	1.0	1.0
**POST**	−1.5 ± 1	−2.7 ± 1.3	−2.8 ± 1.3	9.45	0.002	0.003	0.00	1.0
**P VALUE**	0.0001	0.02	0.26					
**3. LONG DISTANCE JOURNEY**								
**PRE**	−1.48 ± 1.1	−1.6 ± 1.1	−1.3 ± 1.2	0.39	0.67	1.0	1.0	1.0
**POST**	−1.3 ± 1.15	−1.3 ± 1.3	−1.3 ± 1.1	0.00	0.99	1.0	1.0	1.0
**P VALUE**	0.184	0.326	0.822					
**4. HOLIDAY**								
**PRE**	−1.4 ± 1.3	−1.4 ± 1.2	−1.9 ± 1	1.53	0.22	1.0	0.3	0.4
**POST**	−1.1 ± 0.97	−1.07 ± 1.3	−1.9 ± 1	5.18	0.007	1.0	0.02	0.01
**PVALUE**	0.056	0.050	0.80					
**5. DO PHYSICALLY**								
**PRE**	−4 ± 1.4	−4 ± 1.4	−3.5 ± 1.7	0.84	0.33	1.0	0.78	0.78
**POST**	−2.2 ± 1.4	−3.14 ± 1	−3.7 ± 1.4	10.10	0.001	0.03	0.00	0.24
**P VALUE**	0.0001	0.0001	0.392					
**6. FAMILY**								
**PRE**	−2.4 ± 1.6	−2.5 ± 1.6	−2.2 ± 1.2	0.20	0.81	1.0	1.0	1.0
**POST**	−1.4 ± 1.4	−2.14 ± 1.3	−2.3 ± 1.3	0.58	0.5	1.0	0.8	1.0
**P VALUE**	0.005	0.05	0.822					
**7. SOCIAL LIFE**								
**PRE**	−1.8 ± 1.4	−1.9 ± 1.4	−1.6 ± 1.1	0.40	0.66	1.0	1.0	1.0
**POST**	−1.3 ± 1.15	−1.7 ± 1.4	−1.6 ± 1.1	0.81	0.44	0.73	0.92	1.0
**P VALUE**	0.029	0.05	0.325					
**8. CLOSE PERSONAL RELATIONSHIP**								
**PRE**	−2.1 ± 1.4	−2 ± 1.4	−1.9 ± 1.2	0.14	0.871	1.0	1.0	1.0
**POST**	−1.4 ± 1.2	−1.7 ± 1.4	−1.9 ± 1.2	1.19	0.30	1.0	0.38	1.0
**P VALUE**	0.022	0.04	0.71					
**9. SEX LIFE**								
**PRE**	−2.4 ± 1.4	−2.5 ± 1.4	−2.6 ± 1.8	0.09	0.91	1.0	1.0	1.0
**POST**	−2.3 ± 1.3	−2.3 ± 1.4	−2.7 ± 1.8	1.32	0.27	1.0	0.33	1.0
**P VALUE**	0.026	0.083	0.183					
**10. PHYSICAL APPEARANCE**								
**PRE**	−3.4 ± 1	−3.5 ± 1.17	−3.5 ± 1.6	0.11	0.89	1.0	1.0	1.0
**POST**	−2.4 ± 1.4	−2.9 ± 1.13	−3.6 ± 1.6	5.98	0.003	0.48	0.03	0.16
**P VALUE**	0.0001	0.002	0.325					
**11. SELF CONFIDENCE**								
**PRE**	−3.2 ± 1.03	−2.9 ± 1.13	−2.5 ± 1.3	2.42	0.093	0.94	0.90	0.68
**POST**	−2.3 ± 0.92	−2.6 ± 0.95	−2.5 ± 1.2	0.50	0.60	0.97	1.0	1.0
**P VALUE**	0.0002	0.005	0.325					
**12. MOTIVATION**								
**PRE**	−2.3 ± 1.18	−2.2 ± 1.2	−2.06 ± 1	0.34	0.71	1.0	1.0	1.0
**POST**	−1.8 ± 1.22	−2 ± 1.12	−2.03 ± 1	0.16	0.85	1.0	1.0	1.0
**P VALUE**	0.004	0.009	0.744					
**13. PEOPLE GENERAL REACTION**								
**PRE**	−2.02 ± 1.4	−2.02 ± 1.4	−1.7 ± 1.2	0.35	0.70	1.0	1.0	1.0
**POST**	−1.76 ± 1.3	−1.85 ± 1.2	−1.7 ± 1.1	0.10	0.90	1.0	1.0	1.0
**P VALUE**	0.09	0.017	0.325					
**14. FUTURE**								
**PRE**	−3.2 ± 1.8	−3.2 ± 1.8	−3.6 ± 1.4	0.66	0.52	1.0	0.8	1.0
**POST**	−2.5 ± 1.5	−2.7 ± 1.6	−3.7 ± 1.2	5.64	0.005	1.0	0.07	0.41
**P VALUE**	0.003	0.015	0.521					
**15. FINANCIAL SITUATION**								
**PRE**	−3.8 ± 1.07	−3.9 ± 1.4	−3.96 ± .8	0.04	0.957	1.0	1.0	1.0
**POST**	−3.2 ± 1.24	−3.4 ± 1.4	−4.1 ± 0.7	6.10	0.003	1.0	0.04	0.04
**P VALUE**	0.002	0.0032	0.089					
**16. LIVING CONDITION**								
**PRE**	−2.3 ± 1.3	−2.4 ± 1.3	−2.2 ± 0.8	0.18	0.832	1.0	1.0	1.0
**POST**	−1.6 ± 1.37	2.2 ± 1.4	−2.1 ± 0.8	2.17	0.12	0.18	0.28	1.0
**P VALUE**	0.001	0.083	0.263					
**17. DEPENDENCE**								
**PRE**	−2 ± 1.08	−2 ± 1.08	−1.96 ± 1	0.01	0.99	1.0	1.0	1.0
**POST**	−1.5 ± 1	−1.6 ± 1.09	−2.5 ± 1.2	2.62	0.07	0.18	0.28	1.0
**P VALUE**	0.011	0.043	0.056					
**18. FREEDOM TO EAT**								
**PRE**	−4.8 ± 1.04	−4.9 ± 1.1	−4.6 ± 0.9	0.77	0.46	1.0	1.0	1.0
**POST**	−4.1 ± 1.06	−4.3 ± 0.98	−4.6 ± 0.9	2.72	0.071	1.0	0.07	0.48
**P VALUE**	0.002	0.001	0.325					
**19. FREEDOM TO DRINK**								
**PRE**	−2.5 ± 1.47	−2.7 ± 1.24	−3 ± 1.24	0.12	0.88	1.0	1.0	1.0
**POST**	−2.5 ± 1.47	−2.7 ± 1.24	−3 ± 1.24	1.28	0.28	1.0	0.36	0.93
**P VALUE**	-	-	-					

**Figure 1:**
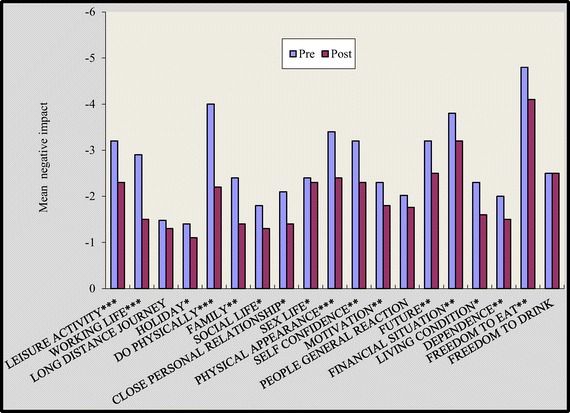
**Impact of diabetes on individual life domains before and after intervention for group A.** *p<0.05, *p<0.01, *p<0.001. p value indicate significance of difference in group A *p<0.05, **p<0.01, ***p<0.001.

**Figure 2:**
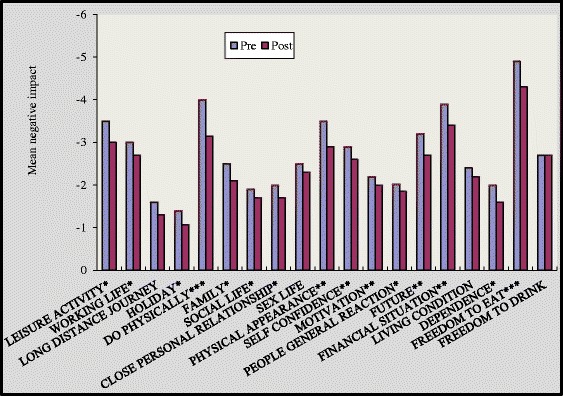
**Impact of diabetes on individual life domains before and after intervention for group B.** p value indicates significance of difference in group B *p<0.05, **P<0.01, ***p<0.001.

**Figure 3:**
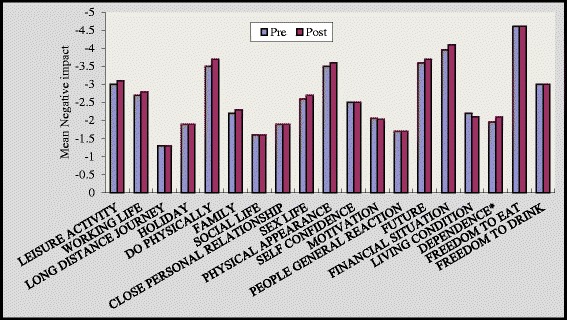
**Impact of diabetes on individual life domains before and after intervention for group C.** *p<0.05, *p<0.01, *p<0.001. p value indicate significance of difference in group C *p<0.05.

On multiple comparisons at baseline, non significant differences were found in between three groups for all the nineteen items of quality of life. Though, after 16 weeks we found that group A and group B showed statically significant reduction in negative impact of ‘quality of life domains’ [Figures 1 and 2]. In group A significant improvement was noted in all domains of quality of life except long distance journey [Figure 1] (p < 0.05). In the group B participant’s experienced significant improvement in all domains except long distance journey, sex life and living condition [Figure 2]. Responses to quality of life domains were more negative amongst control group [Figure 3] compared to group A and group B after 16 weeks of study.

**Figure 4:**
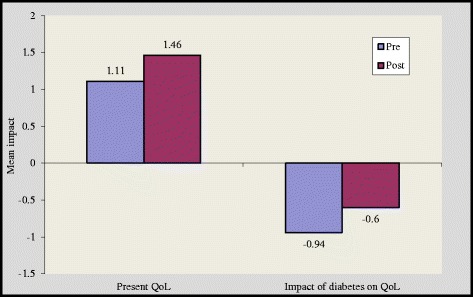
**Impact of diabetes before and after intervention in group A.**

**Figure 5:**
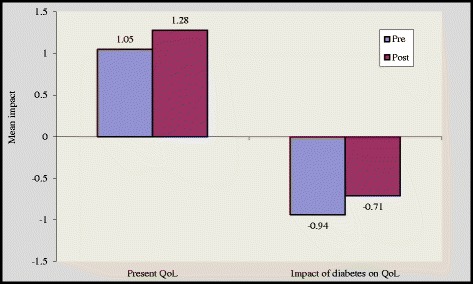
**Impact of diabetes before and after intervention in group B.**

**Figure 6:**
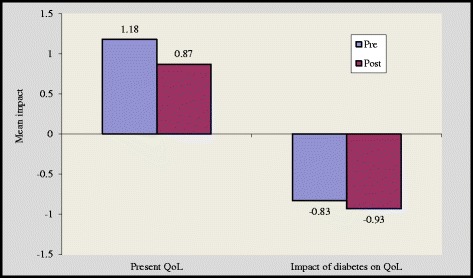
**Impact of diabetes before and after intervention in group C.**

There was a significant improvement in overview item 1(OV1) and overview item 2 (OV 2) scores by 1.11 ± 0.79 to 1.46 ± 0.50 (p < 0.05) and 1.05 ± 0.8 to 1.28 ± 0.46 (p < 0.05) in group A and group B respectively [Figures 4 and 5]. However, subjects in the control group reported higher negative impact of diabetes on quality of life after 16 weeks [Figure 6]. We noticed that the item “Freedom to eat” had highest negative impact among all subgroups. Other domains that were adversely affected by diabetes are ‘leisure activity, ‘do physically’, ‘physical appearance’, ‘self confidence’, ‘future’ and ‘financial situation’.

There were changes in general well being (GWB) scores in both the interventional as well as control group. In terms of percentage there was significant increase in overall well being scores by 43% and 19.2% in group A and group B respectively(p < 0.001) [Table 3]. Repeated measures ANOVA have shown significant results after 16 weeks among all the variables of GWB.

**Table 3:** **Pre intervention and post intervention impact of diabetes on well being**

**General well being**				**Post hoc (Bonferroni)**				
	**GROUP-A**	**GROUP-B**	**GROUP-C**	**ANOVA**		**A and B**	**A and C**	**B and C**
**1. NWB**				F	P			
**PRE**	5.5 ± 3.2	5.5 ± 3.2	6.7 ± 2.7	1.64	0.19	1.0	0.36	0.34
**POST**	2.3 ± 2.3	3.7 ± 2.7	7.3 ± 2.7	29.6	0.0001	0.14	0.00	0.00
**P VALUE**	0.0001	0.0001	0.003					
**2. Energy**								
**PRE**	6.7 ± 1.7	6.9 ± 2.12	5.9 ± 2.7	1.85	0.16	1.0	0.47	0.20
**POST**	8.9 ± 1.5	8.5 ± 1.4	5.3 ± 2.8	29.5	0.0001	1.0	0.00	0.00
**P VALUE**	0.0001	0.0001	0.003					
**3. PWB**								
**PRE**	7.08 ± 2.7	8.5 ± 1.7	7.6 ± 1.9	3.82	0.025	0.02	0.80	0.35
**POST**	10.3 ± 1.1	9.4 ± 1.3	6.7 ± 2	44.61	0.0001	0.11	0.00	0.00
**P VALUE**	0.0001	0.0001	0.0001					
**4. GWB**								
**PRE**	20.2 ± 7.4	21.8 ± 5.3	18.9 ± 5.6	1.87	0.15	0.81	1.0	0.17
**POST**	28.9 ± 4.3	26 ± 4	16.6 ± 5.6	56.02	0.0001	0.08	0.00	0.00
**P VALUE**	0.0001	0.0001	0.0001					

NWB: Negative wellbeing; PWB: Positive wellbeing; GWB: General wellbeing.

Post hoc comparison revealed that changes observed for ‘leisure activity’, ‘working life’ and ‘do physically’ were more significant in between self reported and supervised group (p < 0.05). It has been found that the comparisons in between groups A and B, A and C for ‘leisure activity’, ‘working life’ and ‘do physically’ are highly significant [Table 2]. This indicates that the subjects undergoing intervention in group A showed significant faster improvement in these parameters as compared to group B and group C.

**Figure 7:**
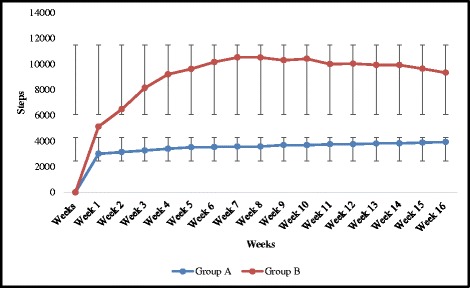
**Weekly average step counts in Group A and Group B.**

Subjects in both experimental groups (supervised and self reported group) had low physical activity at week 1. Subjects in the supervised group reported higher physical activity in comparison to their week 1 activity (3012 ± 194) to (3934 ± 207) after 16 weeks. In self reported group, subjects showed marginal decline in their step count after 10 weeks but overall step count was higher than that at week 1 [Figure 7].

## Discussion

The present study was designed to examine effectiveness of supervised and self reported pedometer based ambulatory protocols on quality of life and well being among individuals with T2DM. Quality of life issues have been largely ignored in the Indian context to the best of our knowledge; no other study has shown the impact of pedometer based walking intervention on quality of life in Asian Indians. Many previous studies have focussed on physical activity responses on physiological parameters, but little attention has been paid on psychological variables among type 2 diabetes individuals. The study explicitly determines influence of motivation in target oriented approach and its effect on quality of life of T2DM individuals.

Quality of life measures are used throughout medicine to evaluate health care outcomes, particularly in chronic conditions, where significant health difficulties may persist even after treatment [24]. Nineteen diabetes specific quality of life domains used in current study address projected social, physical and emotional functioning [25]. Use of ADDQoL would help health care professionals and diabetologists to gain information about the psychological effects of diabetes on different domains of quality of life and will also help to determine influence of physical activity on social, physical and emotional functioning. The results of our study aimed to address the questions described above in background. In the present study, the majority of the participants rated negative impact of diabetes on all the domains. Most severely impacted domains by diabetes were ‘leisure activity’, ‘do physically’, ‘physical appearance’, ‘self confidence’, ‘future worries’, ‘financial situation’ and ‘freedom to eat’. Results of the baseline readings indicated that diagnosis and management of diabetes puts participants under psychosocial challenges.

For the entire sample of participants, the most negatively impacted item was ‘Freedom to eat’. Results of the present study are consistent with the past literature. In many previous studies using ADDQoL ‘freedom to eat as I wish’ was the most negatively impacted domain [10]. Collin’s et al. [26] found the impact of diabetes on quality of life with particular reference to the effects of freedom to eat, enjoyment of food, freedom to drink and worries about the future. Adepu et al. [27] found that, most commonly affected domains of quality of life were freedom to eat, freedom to drink and enjoyment of food followed by family life, sex life, ease to travel, working life and finances.

The magnitude of improvement was higher in supervised group as in this group 17 out of 19 domains showed substantial improvement while in self reported group 15 domains showed such an improvement [Figures 1 and 2]. Two overview items used in this study provide global measures of individual ‘present quality of life ’and the ‘diabetes specific QoL’. In our study, at the end of 16 weeks 6 respondents reported better quality of life in group A while in group B only 3 respondents reported better quality of life [Figures 4 and 5].

We also noticed significant impact of diabetes on “financial worries”. Such prominent impact on ‘financial worries’ domain in India may be attributed to weak health system and poor insurance coverage [28]. Previous studies have shown negative impact of medication on quality of life and overmedication may also impact medication taking behaviour, expenses and can also lead to side effects [29]. It is highly plausible that fear of developing complication can impair quality of life and thus affect ‘future worries’ domain in the present sample. During 16 weeks of the study period, we found improvement broadly in all domains while no change was observed in domain ‘freedom to drink’.

Use of pedometers has been recommended for physical activity interventions to motivate individuals leading to improvement in their ambulatory physical activity [30]. Findings of present study showed that pedometer based progressive walking induced favourable changes in ‘low active’ adults over a period of 16 weeks. Overall, group A participants showed an increase in average step counts of 922/30–40 min/session and group B participants showed an increase in average step counts of 4208/day after 16 weeks. We believe it’s worthy to notice these changes as we found improvement in quality of life after the increase in their number of steps at 16 weeks. We have observed improvement in both the groups, though the target approach for both the groups was different. Setting a step goal for step counts and the use of step diary may have proved to be key motivational factors for increasing physical activity [31]. The motivation factor of using pedometer to achieve step count targets makes exercise session more interesting which in turn leads to beneficial changes on a person’s wellbeing and quality of life.

The results of present study reported highly significant changes in leisure activity, working life, do physically, physical appearance, self confidence, motivation, living condition and all the variables of wellbeing in the supervised group (p < 0.001) [Figure 1] [Table 2]. Participants in the group A and group B experienced a significant improvement in “do physically” item by 45% in group A and 22.5% in group B, while control group experienced non significant decline in “do physically” domain by 5.7%. Pedometers motivated patients to take more steps and helped them to step into a healthy life style. Impact of diabetes on quality of life was remained unchanged among control group who did not participate in any physical activity intervention. Supervised environment might have strengthened sense of awareness in participants and also might have helped them to maintain their standard activity throughout the study protocol. Though significant improvement was noted in self reported group but they couldn’t maintained their physical activity till the end of the study. Other key areas which might have led to improvement in supervised group were regular contact with the participants, supervision of desired goals and motivation to participants which eventually improved their self confidence.

Improved quality of life has been linked to important clinical parameters of reduced morbidity and mortality in a variety of chronic conditions and to reduced health care expenditure [32]. Improving physical wellbeing may also lead to improved psychological well being and it’s generally accepted that physical activity may have positive effects on mood and anxiety [33]. Psychological wellbeing is an important factor for people with diabetes and an important measurement parameter for health care providers. Patient’s emotional and psychological wellbeing is important and same needs to be monitored while diabetes-care. Poor well-being impedes diabetes self-care, adaptational tasks in chronic illness (coping), maintenance of emotional balance after diagnosis (loss of health, self-esteem), coping with physical complaints and functional limitations, maintenance of social roles and coping with negative labelling (stigma) [34].

In the current study, the percentage change for NWB was 58% and 32% decrease in group A and group B respectively. The percentage change for energy and PWB was increases by 32%, 45% respectively in-group A and 23%, 10% for group B respectively. Percentage improvement in group B is relatively lower by which it was hypothesized that group A benefitted by regular monitoring. Improvement in all the 3 parameters (PWB, NWB and energy) was seen in the both experimental groups, though scores of group A were slightly higher than that of group B. Contribution of exercise in enhancing PWB and energy The positive and important role of exercise in enhancing the PWB and energy cannot be ignored as its equally important as any other physiological parameter.

As a conclusion we may state that, pedometer determined activity has the potential to improve the quality of life. Motivation with pedometer under the supervision is helpful in achieving target step counts and improves quality of life. The results of this study clearly imply that 30–40 min/day/session of moderate intensity walking with pedometer is an effective method for the sedentary T2DM individuals to enhance quality of life.
